# Supplementary material for: Isolation of MERS-related coronavirus from lesser bamboo bats that uses DPP4 and infects human-DPP4-transgenic mice
Source: Nat Commun. 2021 Jan 11;12:216. doi: 10.1038/s41467-020-20458-9 (PMC7801609; doi:10.1038/s41467-020-20458-9)
Supplement: Supplementary file 1 — Supplementary Information [file 41467_2020_20458_MOESM1_ESM.pdf]

Supplementary Information

Isolation of MERS-related coronavirus from lesser bamboo bats that uses DPP4 and infects human-DPP4-transgenic mice

Susanna K. P. Lau*†, Rachel Y. Y. Fan*, Longchao Zhu*, Kenneth S. M. Li*, Antonio C. P. Wong, Hayes K. H. Luk, Emily Y. M. Wong, Carol S. F. Lam, George C. S. Lo, Joshua Fung, Zirong He, Felix C. H. Fok, Rex K. H. Au-Yeung, Libiao Zhang, Kin-Hang Kok, Kwok-Yung Yuen* and Patrick C. Y. Woo†

*These authors contributed equally to this work.

†Corresponding authors. Email: pcywoo@hku.hk (P.C.Y.W.); skplau@hku.hk (S.K.P.L.)

Supplementary Tables 1 -9

Supplementary Figures 1 -5

Supplementary Table 1 RT-PCR detection of *Ty*-BatCoV HKU4 from bat samples

Scientific name	No. of bats tested	No. of bats positive for *Ty*-BatCoV HKU4	Sampling location of bats
Aselliscus stoliczkanus	55	0	Yunnan
Chaerephon plicata	31	0	Hainan, Yunnan
Cynopterus sphinx	86	0	Guangdong, Guangxi, Yunnan, Hainan, Hong Kong
Eonycteris spelaea	53	0	Yunnan
Hipposideros armiger	219	0	Guangdong, Guangxi, Guizhou, Yunnan, Hong Kong
Hipposideros larvatus	48	0	Guangdong, Guangxi, Hainan, Guizhou
Hipposideros pomona	774	0	Guangdong, Hainan, Guizhou, Hong Kong
Hipposideros pratti	19	0	Guangdong
Hypsugo pulveratus	6	0	Guangdong, Hong Kong
Ia io	26	0	Guangdong, Yunnan
Kervoula picta	1	0	
Megaderma lyra	1	0	Guangdong
Megaerops ecaudatus	1	0	Yunnan
Miniopterus fuliginosus	30	0	Guangdong, Hainan, Yunnan
Miniopterus magnater	17	0	Hong Kong
Miniopterus pusillus	273	0	Guangdong, Hainan, Hong Kong
Miniopterus schreibersii	492	0	Hong Kong, Yunnan
Molossus sp.	25	0	Yunnan
Myotis altarium	14	0	Guangdong
Myotis chinensis	123	0	Guangdong, Hainan, Hong Kong
Myotis davidii	2	0	Shanxi
Myotis fimbriatus	6	0	Shanxi
Myotis formosus	1	0	Guangxi
Myotis horsfieldii	19	0	Hong Kong
Myotis longipes	4	0	Guangdong
Myotis pequinius	29	0	Shanxi
Myotis ricketti	483	0	Guangdong, Hainan, Hong Kong
Myotis siligorensis	3	0	
Myotis daubentonii	5	0	Guangdong
Nyctalus plancyi	2	0	Guangdong
Nyctalus noctula	14	0	Hong Kong
Pipistrellus abramus	102	0	Guangdong, Hainan, Yunnan, Hong Kong
Pipistrellus .	7	0	Guangdong, Hong Kong
Pipistrellus ceylonicus	1	0	Guangdong
Pipistrellus tenuis	4	0	Hong Kong
Rhinolophus affinis	263		Guangdong, Hainan, Zhejiang, Hong Kong
Rhinolophus ferrumequinum	86	0	Shanxi
Rhinolophus luctus	4	0	Guangdong, Zhejiang
Rhinolophus macrotis	24	0	Guangdong, Guangxi
Rhinolophus pearsoni	29	0	Guangdong, Guizhou
Rhinolophus pusillus	206	0	Guangdong, Guangxi, Hainan, Yunnan, Hong Kong
Rhinolophus rex	4	0	Guangdong
Rhinolophus siamensis	1	0	Guangdong, Yunnan
Rhinolophus sinicus	1004	0	Guangdong, Hainan, Hong Kong
Rhinolophus thomasi	1	0	Yunnan
Rhinolophus sp.	20	0	Yunnan
Rousettus leschenaulti	70	0	Guangdong, Yunnan, Hong Kong
Scotophilus kuhlii	37	0	Guangdong, Hainan, Hong Kong
Taphozous melanopogon	119	0	Yunnan, Guangxi
Tylonycteris pachypus	101	13	Hong Kong
	17	0	Guangzhou, Guangdong
	203	0	Maoming, Guangdong
	193	0	Longzhou, Guangxi
	244	1	Ningming, Guangxi
	271	18	Luodian, Guizhou,
	1	0	Baoting, Hainan
	14	0	Mengla, Yunnan
	1044	32	
Tylonycteris robustula	196	0	Guizhou, Yunnan, Guangxi
Unknown	2	0	
Total	6086	32	

Supplementary Table 2 Cell lines used in this study

Animal species of origin		Site of origin	Abbreviation	Source
Bat	*Hipposideros pomona* (Pomona roundleaf bat)	Kidney	HPK	In-house development
	Miniopterus pusillus (Lesser bent-winged bat)	Kidney	MPK	In-house development
	Myotis ricketti (Rickett's big-footed bat)	Kidney	MRK	In-house development
	Myotis ricketti (Rickett's big-footed bat)	Lung	MRL	In-house development
	Pipistrellus abramus (Japanese pipistrelle)	Kidney	PAK	In-house development
	Pipistrellus abramus (Japanese pipistrelle)	Lung	PAL	In-house development
	Rhinolophus sinicus (Chinese horseshoe bat)	Kidney	RSK	In-house development
	Rhinolophus sinicus (Chinese horseshoe bat)	Lung	RSL	In-house development
	Tylonycteris pachypus (Lesser bamboo bat)	Kidney	TPK	In-house development
	Tylonycteris pachypus (Lesser bamboo bat)	Lung	TPL	In-house development
	Rousettus leschenaultii (Leschenault's rousette)	Kidney	RLK	In-house development
	Rousettus leschenaultii (Leschenault's rousette)	Lung	RLL	In-house development
Camel	*Camelus dromedarius* (Arabian camel)	Kidney	Caki-3-R	CCLV-RIE 1284
	Camelus dromedarius	Skin	Dubca	ATCC CRL-2276
Human	*Homo sapiens*	Lung	Calu-3	ATCC HTB-55
	Homo sapiens	Cervix	HeLa	ATCC CCL-2
	Homo sapiens	Colon	HRT-18G	ATCC CRL-11663
	Homo sapiens	Colon	HT-29	ATCC HTB-38
	Homo sapiens	Liver	Huh-7	JCRB0403
	Homo sapiens	Histiocytes	His-1	In-house development
	Homo sapiens	Bronchia/trachea	NHBE	Lonza CC-2540
	Homo sapiens	Lung	A549	ATCC CCL-185
	Homo sapiens	Cervix	Hep-2	ATCC CCL-23
	Homo sapiens	Embryonic Lung	HFL	In-house development
	Homo sapiens	Neuromuscular	NT2/D1	ATCC CRL-1973
	Homo sapiens	Muscle	RD	ATCC CCL-136
	Homo sapiens	Peripheral blood	THP-1	ATCC TIB-202
	Homo sapiens	Lymphoblast	Raji	ATCC CCL-86
	Homo sapiens	Embryonic Kidney	293T	ATCC CRL-3216
	Homo sapiens	Colon	CaCO2	ATCC HTB-37
Cat	*Felis catus*	Kidney	CRFK	ATCC CCL-94
Pig	*Sus scrofa*	Kidney	PK15	ATCC CCL-33
Mouse	*Mus musculus*	Embryonic kidney	3T3	ATCC CCL-92
Rat	*Rattus norvegicus*	Kidney	RK3E	ATCC CRL-1895
Monkey	*Macaca mulatta* (Rhesus monkey)	Kidney	LLC-MK2	ATCC CCL-7
	Cercopithecus aethiops (African green monkey)	Kidney	Vero	ATCC CCL-81
	Cercopithecus aethiops (African green monkey)	Kidney	Vero E6	ATCC CRL-1586
	Cercopithecus aethiops (African green monkey)	Kidney	BS-C-1	ATCC CCL-26

Supplementary Table 3 Neutralization antibody titers of MERS-infected camel sera with MERS-CoV neutralizing antibodies against *Ty*-BatCoV HKU4

Camel sera	Neutralization antibody titer (MERS-CoV)	Neutralization antibody titer (*Ty*-BatCoV HKU4)
1	<10	<10
2	10	<10
3	10	<10
4	10	<10
5	10	<10
6	10	<10
7	10	<10
8	10	<10
9	80	<10
10	80	<10
11	80	<10
12	80	<10
13	80	<10
14	80	<10
15	80	<10
16	160	<10

Supplementary Table 4 Number of mice positive for *Ty*-BatCoV HKU4 SM3A in different tissues

Dpi	Tissues	Number of mice positive
2	lung	4/8
	brain	0/8
	spleen	0/8
4	lung	5/7
	brain	0/7
	spleen	0/7
7	lung	7/11
	brain	5/11
	spleen	1/11
14	lung	0/13
	brain	8/13
	spleen	0/13
28	lung	0/4
	brain	0/4
	spleen	0/4

Supplementary Table 5 List of primers used for complete *Ty*-BatCoV HKU4 S gene sequencing

Primer	Primer sequence (5'->3')	Forward/Reverse (F/R)
LPW 3797	AGATTTATATAAAATTATGGGAA	F
LPW 4102	TACGTGGTTTTAATATGCAATAAAA	R
LPW 3899	TCTCTTACTAATACATCGGCT	F
LPW 3900	AAGACCTGACCATCTTCAGAAA	R
LPW 33089	TTACATTTCTAAGTGTTCTCGC	F
LPW 33090	GTAACAACAATAGGAGAAGTA	R
LPW 2821	GTCATAAAGTGGTGGTAAAACTT	R
LPW 3720	CATTAGTAGTTAGTGATTGTAAA	F
LPW 2320	TTTGGGTAACTCCAATNCCRTT	R
LPW 2319	ATTAATGCTAGAGAYCTHMTTTG	F
LPW 33092	AGAGACCATAGGCTGCTGTAA	R
LPW 33091	CTGGTTTATCATCCTTTGCTG	F
LPW 2824	TTTGCCGCTATACCTTTTGCACAA	F
LPW 2317	GAGCCAAACATACCANGGCCAYTT	R
LPW 33093	TTTTCAAGAATGTTACCTCGC	F
LPW 33094	ATACGGAAAAACCAACATCA	R

Supplementary Table 6 List of primers used for complete genome sequencing of *Ty*-BatCoV HKU4 SM3A

Primer	Primer sequence (5'→3')	Forward/Reverse (F/R)
LPW4830	TCCAAACTTGAATCATATGGAAA	F
LPW4519	AACTTAAATAATAGCCTTGCTGTGT	R
LPW35132	TACTGGAGGTATGCTTTCAA	F
LPW4632	TATATGTGTGGCAAAAACGGTAAA	F
LPW4729	TGGGTTGTTGTGATGGTACTAT	F
LPW4633	ATGCTCATTAGTTGGTTCTAATTGA	R
LPW4469	AGTGCTAGGGGTAGAGTCTATTGA	R
LPW4503	AGTAATACGGAATCACCTACATGAA	R
LPW4502	TGGTGGTGATGTTCAGGAAGAAT	F
LPW4497	TACAACAAATTTCCACTGCTGAA	R
LPW4429	AGGTGAATATTTAATGGTAGGTTTA	R
LPW4930	TATGTCCATATTCGAGTGAAGGAT	F
LPW4931	TAGCTTTCCAGTCTACACGCTTA	R
LPW4498	TTATGGCCTAAGAAACAACCAA	F
LPW4737	ACGTGTTGAAGCTTCCACCATT	F
LPW4831	TTCATATAATCACCACTATTCCAAA	R
LPW4430	TTTAGGTTGTTTGATTGACCTTA	F
LPW4829	TTCTCTGATAGTGGCTGTGTGCTTT	F
LPW4500	TGCGGCACAAGCCTCTCTATA	R
LPW35133	GCTTTCAGGATGCTGCTAG	F
LPW3796	TCTGGTGGGTTTTACAAACCACTT	R
LPW3794	ACTTACAACCATTTAGAACTG	R
LPW3793	TCTATATGCATCAGATGGAATTA	F
LPW3795	ATCAAATGGTACACATACTGGTA	F
LPW35134	CGCACTACACATTTGGACT	F
LPW3790	AACATCATTATCTAACTTCTTAA	R
LPW4504	AGCTATGACATCTATGTATAAGCAA	F
LPW4072	TTCTAGTCGGGCATTTACACTAGAA	R
LPW3232	AACTAATATGCTCTTTAACACTTCAC	R
LPW3283	GTAATGTCTGTCAGTATTGGGTT	F
LPW2773	GTTGGGTAATAACAAAATCACCAA	R
LPW2771	TGYTAYGCTTTAMGNCAYTTYGA	F
LPW2626	GTTTTAACACTYGATAAYGARGA	F
LPW2630	AGTATATTGAARTTNGCACARTG	R
LPW2738	CCACCCTAATTGTGTTAATTGTA	F
LPW2775	TAACTGAAGACCCTTCCTTGAAA	R
LPW3234	GCCAAAATCAATGACGCTAAAAT	R
LPW3233	GGCAATTTTAATAAAGATTTTTATGA	F
LPW1507	GGTTGGGACTATCCTAAGTGTGA	F
LPW8143	ACCATCATCNGANARDATCATNA	R
LPW1037	WTATKTKAARCCWGGTGG	F

LPW1040	KYDBWRTTRTARCAMACAAC	R
LPW3235	CTTAATAAACACTTTTCTATGATGAT	F
LPW2583	AAGTGTTGTTATGATCAYGTNAT	F
LPW2587	TTACCTCTACCAGGAGGNCCYTG	R
LPW2679	CAGCACGCATTGTTTATACAGCAT	F
LPW2774	TTAGTCCTAACATGGATCTAGCT	R
LPW36404	AAAGGCAGTGTGACGCA	F
LPW2739	AGCTAGATCCATGTTAGGACTAA	F
LPW2595	TGTATCCCCACTGYTGDATRTC	R
LPW2598	CAATCCACATTRCARTTRCARAA	R
LPW2820	CAACCACGACCGGTATTGTGGCAT	F
LPW2940	TTAAGAAATTGTTTTACACAGAT	F
LPW2934	ATCTCCATAAACGATRTGYTCRAA	R
LPW3710	ACAACATCTATGGTACAACTACAA	F
LPW3798	TCCAAAATATATAATTGGCA	R
LPW4102	TACGTGGTTTTAATATGCAATAAAA	R
LPW3797	AGATTTATATAAAATTATGGGAA	F
LPW3899	TCTCTTACTAATACATCGGCT	F
LPW3900	AAGACCTGACCATCTTCAGAAA	R
LPW3712	CTAGCGCTATAACTTCTAAAAGTA	R
LPW4103	TGGTGCAAACCAAGATGTTGAAA	F
LPW3720	CATTAGTAGTTAGTGATTGTAAA	F
LPW2821	GTCATAAAGTGGTGGTAAAACTT	R
LPW2319	ATTAATGCTAGAGAYCTHMTTTG	F
LPW2320	TTTGGGTAACTCCAATNCCRTT	R
LPW2824	TTTGCCGCTATACCTTTTGCACAA	F
LPW2317	GAGCCAAACATACCANGGCCAYTT	R
LPW36405	TTG ACC TTT CAG ATG AGA TG	F
LPW36406	TGG ATA CAG GTA ATG ATA CTG AC	R
LPW4394	GGGGAGCATACCAAGAAACATT	R
LPW36407	TCAGCAAGAACCTACTCACT	F
LPW4486	TTGGAGTTTAAAAAGCTCTGTGTT	F
LPW4487	AAGGGGTACTGTTTTATCACTAGAA	R
LPW4380	GACCCTAAAGTGTCTTTTGAGTA	R
LPW2918	TTGATGTGGCTTAGCTAYTTYGT	F
LPW2831	TGGGCCAGTTCCTARRTARTARAA	R
LPW3815	GAAAATCAACACCGGTAATGGT	F
LPW418	GACCACGCGTATCGATGTCGACTTTTTTTTTTTTTTTTV	R

Supplementary Table 7 List of primers used for cloning

Gene	Primer	Sequence (5'-3')
dcDPP4	F: LPW34010	ATTC**CTCGAG**ATGAAGACACCGTGGAGGGTGCTCCTGGGA
	R: LPW34011	ATT**GGTACC**CTAAGGTAAAGAGAAGCATTGCTTTAGGAAGTG
TpDPP4	F: LPW33360	ATTC**CTCGAG**AGTCGCAGGACCTATACCCTAACTGATTATTTAAAAAGT
	R: LPW33361	ATT**GGTACC**TAAGGTAAAGAGAAGCATTGCTTTAGGAAGTGGGTCATG
hDPP4	F: LPW34048	ATTC**CTCGAG**ATGAAGACACCGTGGAAGGTTCTTCTGG
	R: LPW34039	ATT**GGTACC**AGGTAAAGAGAAACATTGTTTTATGAAGTG
HA tag	F: LPW34039	ATT**GGTACC**TACCCATACGATGTTCCAGATTACGCTTAG**GCGGCCGC**ACT
	R: LPW34040	AGT**GCGGCCGC**CTAAGCGTAATCTGGAACATCGTATGGGTA**GGTACC**AAT
MERS-RBD-Fc-his	F: LPW36700	ATCC**GCGGCCGC**GCCACCATGAAAATGATACACTCAGTGTTTCTACTGATGTTC
	R: LPW36701	TGTC**ACTAGT**ATATTCCACGCAATTGCCTAATTGAGAGGCAATTTTTGTGTCAT
HKU4-RBD-Fc-his	F: LPW36702	ATCC**GCGGCCGC**GCCACCATGAAAATGATTCACTCAGTGTTCTTGCTGATGTTT
	R: LPW36703	TGTC**ACTAGT**GTAATCGACACATTTCCCCAGTCTGTTTGTGATGGTGAGAGAGTC

Notes: the restriction site was highlighted with bold and underlined.

Supplementary Table 8 Gating strategy used for flow cytometry analysis

HEK293T cell	Calibration for negative control
HEK293T-hDPP4	Alexa Fluor 647 conjugated anti-HA antibody<br>6x-His Tag Monoclonal Antibody FITC
HEK293T-hDPP4+MERS-RBD	Alexa Fluor 647 conjugated anti-HA antibody<br>6x-His Tag Monoclonal Antibody FITC
HEK293T-hDPP4+HKU4-RBD	Alexa Fluor 647 conjugated anti-HA antibody<br>6x-His Tag Monoclonal Antibody FITC
HEK293T-*Tp*DPP4	Alexa Fluor 647 conjugated anti-HA antibody<br>6x-His Tag Monoclonal Antibody FITC
HEK293T-*Tp*DPP4+MERS-RBD	Alexa Fluor 647 conjugated anti-HA antibody<br>6x-His Tag Monoclonal Antibody FITC
HEK293T-*Tp*DPP4+HKU4-RBD	Alexa Fluor 647 conjugated anti-HA antibody<br>6x-His Tag Monoclonal Antibody FITC
HEK293T-dcDPP4	Alexa Fluor 647 conjugated anti-HA antibody<br>6x-His Tag Monoclonal Antibody FITC
HEK293T-dcDPP4+MERS-RBD	Alexa Fluor 647 conjugated anti-HA antibody<br>6x-His Tag Monoclonal Antibody FITC
HEK293T-dcDPP4+HKU4-RBD	Alexa Fluor 647 conjugated anti-HA antibody<br>6x-His Tag Monoclonal Antibody FITC

Supplementary Table 9 List of primers used for mouse cytokine profiling

Gene	Primer	Sequence (5'-3')
IL-1β	F: LPW34068	TGGACCTTCCAGGATGAGGACA
	R: LPW34069	GTTCATCTCGGAGCCTGTAGTG
IL-2	F: LPW34070	GGACTTTCTGAGGAGATGGATAG
	R: LPW34071	TGTTGTAAGCAGGAGGTACATAG
IL-6	F: LPW36745	TAGTCCTTCCTACCCCAATTTCC
	R: LPW36746	TTGGTCCTTAGCCACTCCTTC
IL-12p40	F: LPW34074	TTGAACTGGCGTTGGAAGCACG
	R: LPW34075	CCACCTGTGAGTTCTTCAAAGGC
IFN-β	F: LPW34090	GCGGACTTCAAGATCCCTATG
	R: LPW34091	ACAATAGTCTCATTCCACCCAG
IFN-γ	F: LPW34092	AAATCCTGCAGAGCCAGATTAT
	R: LPW34093	GCTGTTGCTGAAGAAGGTAGTA
IP-10	F: LPW34517	ATCAGCACCATGAACCCAAG
	R: LPW34518	GTGGCAATGATCTCAACACG
MCP-1	F: LPW36689	ACCACAGTCCATGCCATCAC
	R: LPW36690	TTGAGGTGGTTGTGGAAAAG
MIP-1α	F: LPW34086	ACCATGACACTCTGCAACC
	R: LPW34087	CGATGAATTGGCGTGGAATC
Mx1	F: LPW36687	GGGGAGGAAATAGAGAAAATGAT
	R: LPW36688	GTTTACAAAGGGCTTGCTTGCT
RANTES	F: LPW34088	CCTGCTGCTTTGCCTACCTCTC
	R: LPW34089	ACACACTTGGCGGTTCCTTCGA
TNF-α	F: LPW34076	TTGTCTACTCCCAGGTTCTCT
	R: LPW34077	GAGGTTGACTTTCTCCTGGTATG
CXCL-1(KC)	F: LPW34084	TCCAGAGCTTGAAGGTGTTGCC
	R: LPW34085	AACCAAGGGAGCTTCAGGGTCA
G-CSF	F: LPW34078	GCAGGCTCTATCGGGTATTTC
	R: LPW34079	CACCCCTAGGTTTTCCATCTG
Mouse β-actin	F: LPW34519	AGAGGGAAATCGTGCGTGAC
	R: LPW34520	CAATAGTGATGACCTGGCCGT

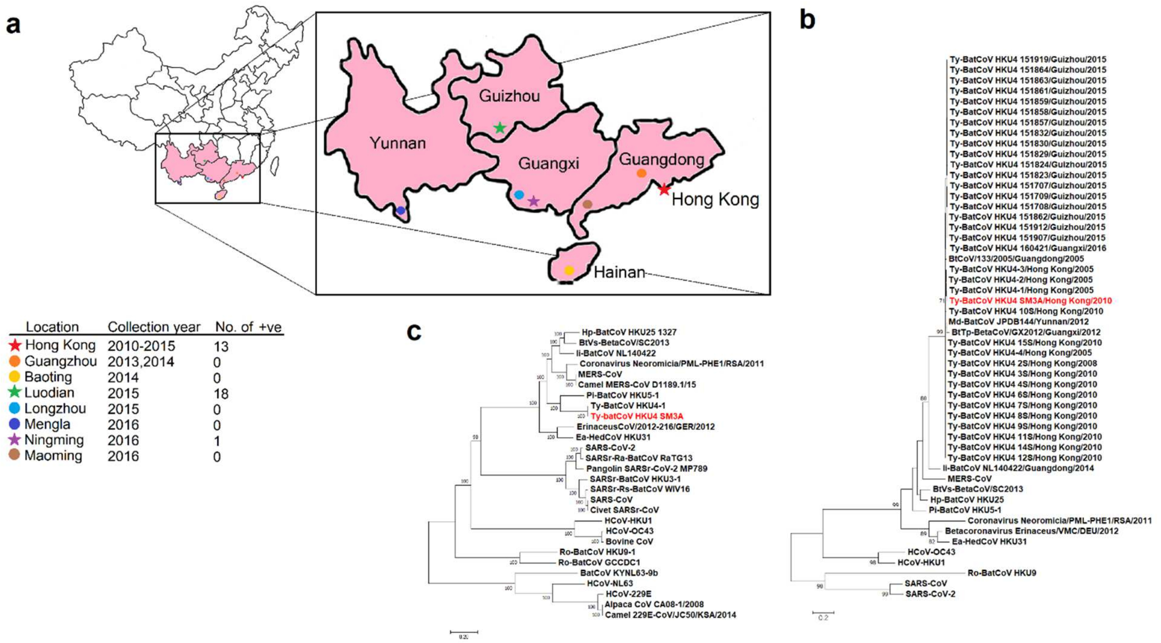

Supplementary Figure 1 A 6-year surveillance study of merbecoviruses in bats from Hong Kong and mainland China. (**a**) Map showing various locations of bat sampling in five provinces of China (Guangxi, Guangdong, Guizhou, Hainan and Yunnan) and Hong Kong. Sampling locations with positive detection of *Ty*-batCoV HKU4 are indicated with stars. (**b**) Phylogenetic analysis of S1 aa sequences of *Ty*-BatCoV HKU4 strains and other betacoronaviruses. The trees were constructed by maximum likelihood method using WAG+G substitution models respectively and bootstrap values calculated from 1000 trees. Only bootstrap values >70% are shown. 175 aa positions were included in the analyses. The scale bars represent 5 substitutions per site respectively. The strain *Ty*-BatCoV HKU4 SM3A is red in color. (**c**) Phylogenetic analysis of the complete genomes of *Ty*-BatCoV HKU4 SM3A and other betacoronaviruses. The trees were constructed by maximum likelihood method using GTR+G+I substitution models respectively and bootstrap values calculated from 1000 trees. Only bootstrap values >70% are shown. The scale bars represent 5 substitutions per site respectively. The strain *Ty*-BatCoV HKU4 SM3A is red in color.

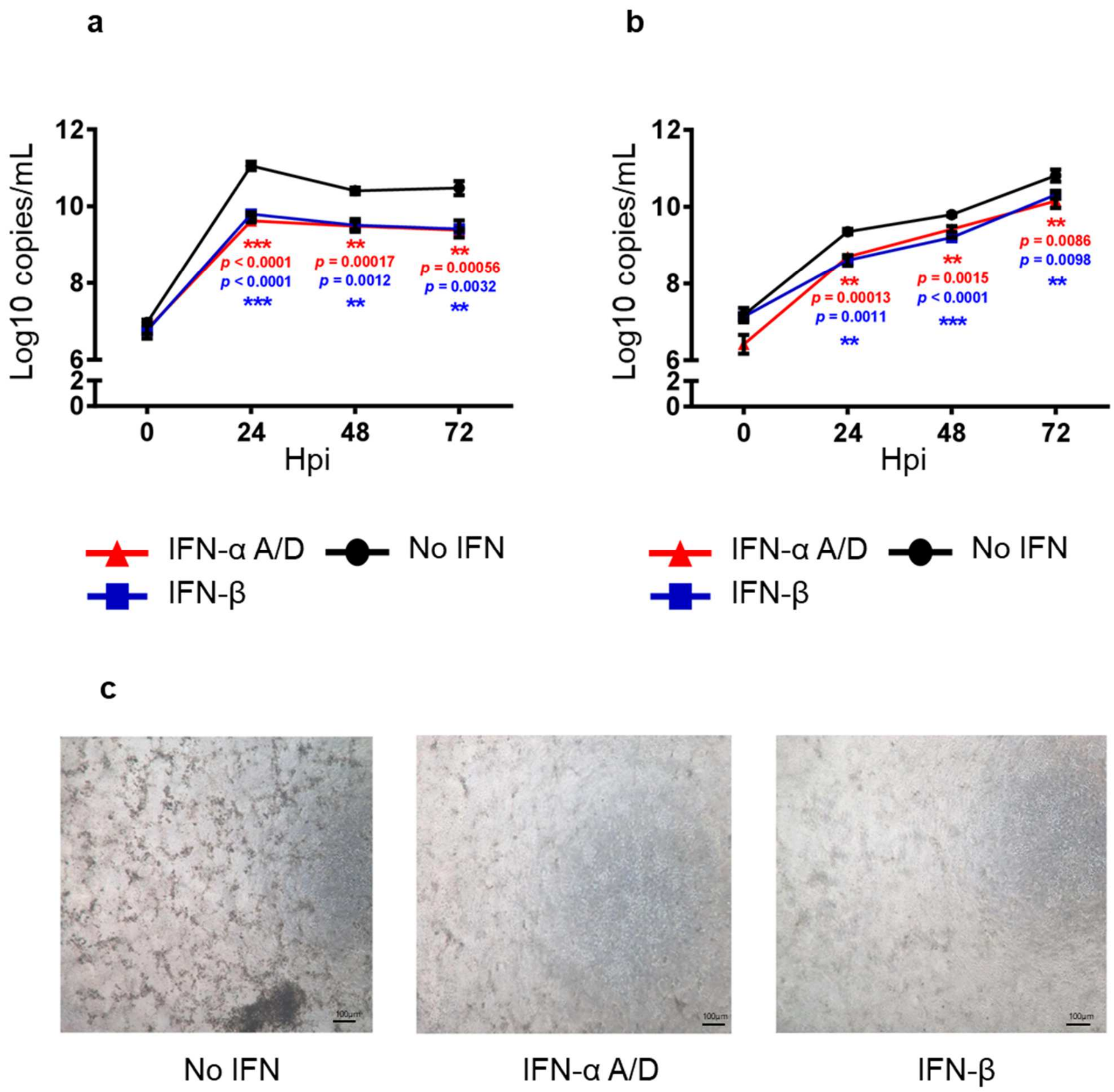

Supplementary Figure 2 *Ty*-BatCoV HKU4 replication is inhibited by interferons-α/β. (**a**) MERS-CoV and (**b**) *Ty*-BatCoV HKU4 SM3A of MOI 1 were inoculated to IFNs pretreated Huh-7 cells. Culture supernatants were harvest at 0, 24, 48, 72 hpi. Viral loads were determined by RT-qPCR. Data are presented as mean values ± SD, n = 3 independent biological replicates at each time point. Dots in each graph represent individual samples. The p-values calculated by multiple two-tailed unpaired t test without correction for multiple comparisons between the control group and different IFN treatment groups at each time point are indicated (Red: IFN-α A/D; Blue: IFN-β). Statistical significances are indicated by the asterisks (*P < 0.05; **P < 0.01; ***P < 0.0001). (**c**) CPE of *Ty*-BatCoV HKU4

infections with different IFNs treatments were observed at 72 hpi. Scale bars, 100 μm. Images are representative of three independent experiments.

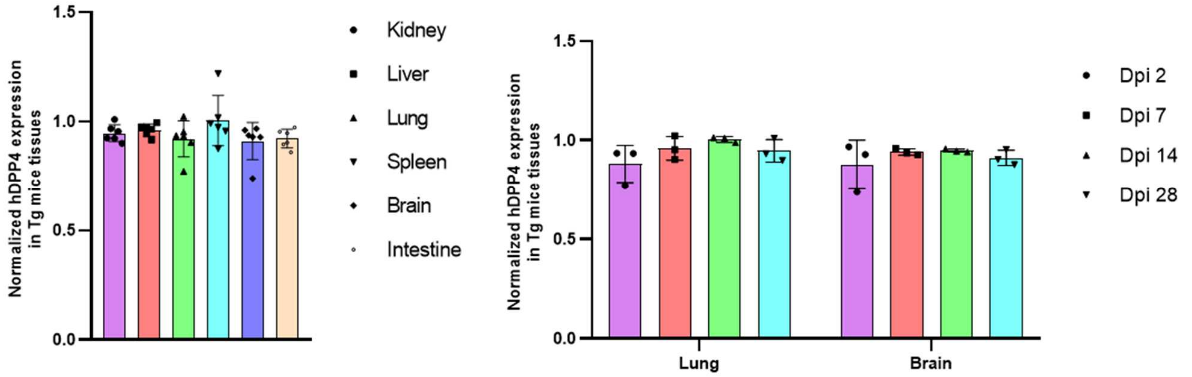

Supplementary Figure 3 Expression of hDPP4 transgene in hDPP4 transgenic mice. (**a**) hDPP4 transgene expression in different tissues of hDPP4 transgenic mice was measured by qRT-PCR normalized to mouse GAPDH. (**b**) hDPP4 transgene expression in lung and brain tissues of hDPP4 transgenic mice from different indicated time points. Data are presented as mean values ± SD, n = 6 (a) or 3 (b) independent biological replicates. Dots in each graph represent individual samples.

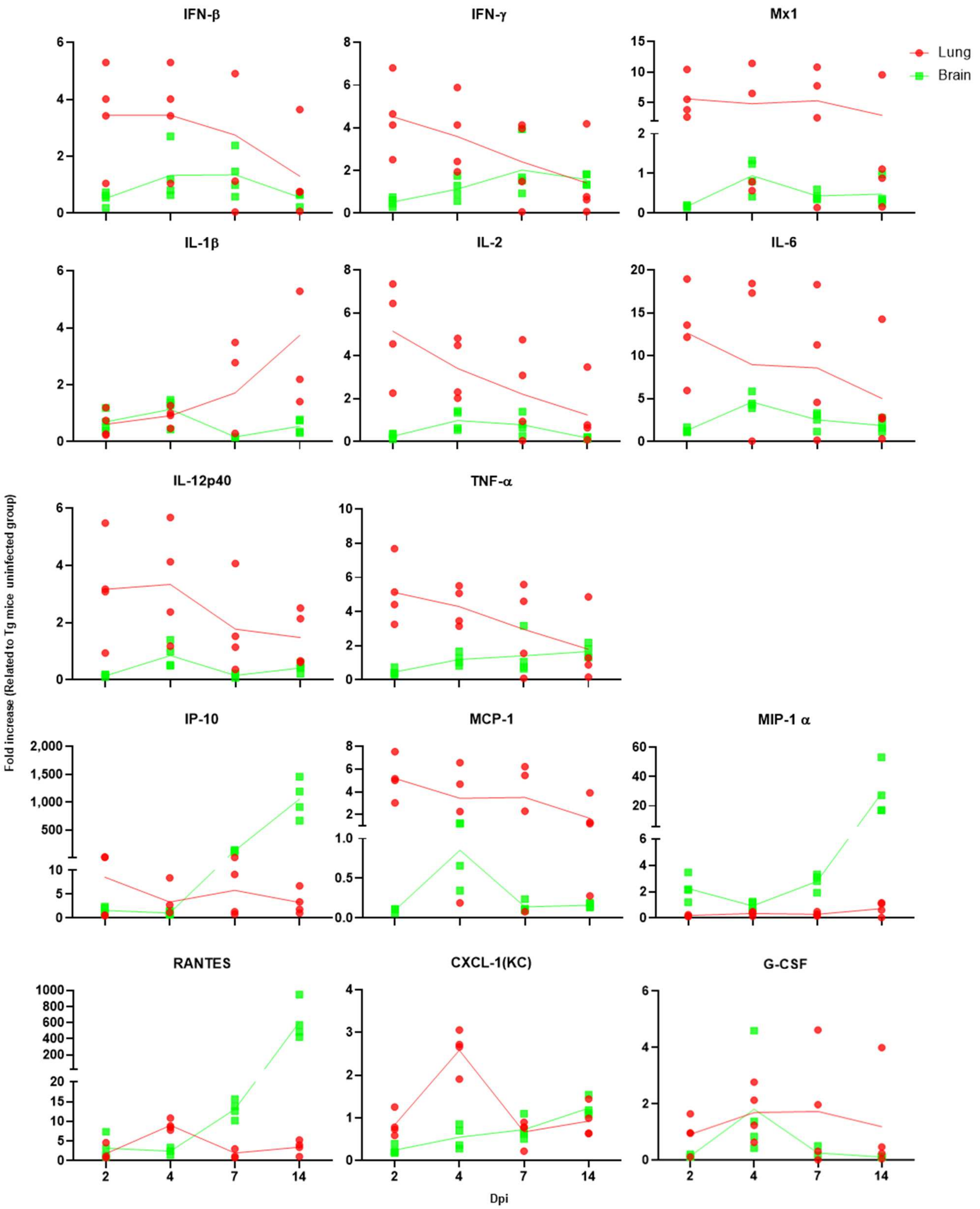

Supplementary Figure 4 Cytokine response in the lung (red) and brain (green) tissues of *Ty*-BatCoV HKU4 SM3A-infected mice. The profiling of innate inflammatory response in the lung and brain tissues of *Ty*-BatCoV HKU4 SM3A-infected and uninfected transgenic mice at indicated time points

was determined by qPCR. The relative expression for each gene was calculated by the comparative ΔΔCT method. Trend lines represent the changes in the relative cytokine level of *Ty*-BatCoV HKU4 SM3A-infected mice in respective tissues (n=4 for each time point).

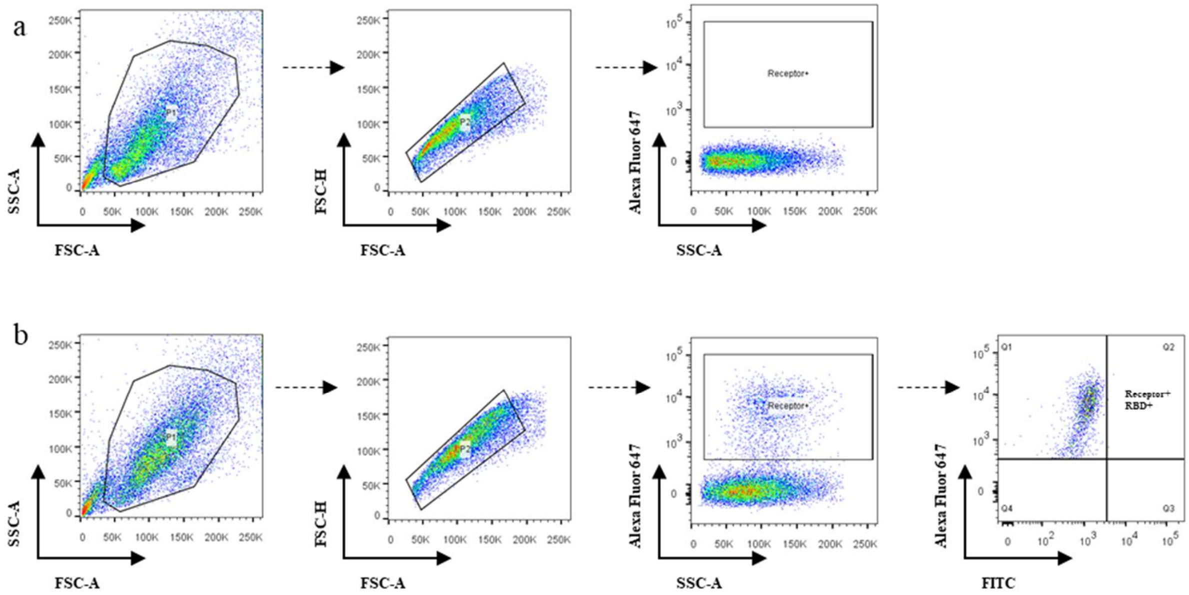

Supplementary Figure 5 Gating strategy used in flow cytometry analysis in Fig. 3a. (**a**) Gating strategy to determine the boundary of different DPP4 receptor-positive (Receptor+) cells by using untransfected 293T cells. (**b**) Gating strategy to determine the boundary of RBD positive (RBD+) cells from Receptor+ cells.
