# Supplementary material for: Isolation of MERS-related coronavirus from lesser bamboo bats that uses DPP4 and infects human-DPP4-transgenic mice
Source: Nat Commun. 2021 Jan 11;12:216. doi: 10.1038/s41467-020-20458-9 (PMC7801609; doi:10.1038/s41467-020-20458-9)
Supplement: Supplementary file 3 — Reporting Summary [file 41467_2020_20458_MOESM3_ESM.pdf]

nature research

Corresponding author(s): Susanna KP Lau and Patrick CY Woo

Last updated by author(s): Nov 18, 2020

Reporting Summary

Nature Research wishes to improve the reproducibility of the work that we publish. This form provides structure for consistency and transparency in reporting. For further information on Nature Research policies, see our Editorial Policies and the Editorial Policy Checklist.

Statistics

For all statistical analyses, confirm that the following items are present in the figure legend, table legend, main text, or Methods section.

n/a	Confirmed	
☐	☒	The exact sample size (*n*) for each experimental group/condition, given as a discrete number and unit of measurement
☐	☒	A statement on whether measurements were taken from distinct samples or whether the same sample was measured repeatedly
☐	☒	The statistical test(s) used AND whether they are one- or two-sided<br>*Only common tests should be described solely by name; describe more complex techniques in the Methods section.*
☒	☐	A description of all covariates tested
☒	☐	A description of any assumptions or corrections, such as tests of normality and adjustment for multiple comparisons
☐	☒	A full description of the statistical parameters including central tendency (e.g. means) or other basic estimates (e.g. regression coefficient) AND variation (e.g. standard deviation) or associated estimates of uncertainty (e.g. confidence intervals)
☐	☒	For null hypothesis testing, the test statistic (e.g. *F*, *t*, *r*) with confidence intervals, effect sizes, degrees of freedom and *P* value noted<br>*Give P values as exact values whenever suitable.*
☒	☐	For Bayesian analysis, information on the choice of priors and Markov chain Monte Carlo settings
☒	☐	For hierarchical and complex designs, identification of the appropriate level for tests and full reporting of outcomes
☒	☐	Estimates of effect sizes (e.g. Cohen's *d*, Pearson's *r*), indicating how they were calculated

Our web collection on statistics for biologists contains articles on many of the points above.

Software and code

Policy information about availability of computer code

Data collection	BD FACSDiva Software, Philips CM100 transmission electron microscope, BIAcore X100, Nikon Eclipse 80i Digital Microscopes, Spot advance computer software (5.1)
Data analysis	BioEdit version 7.2.5, MEGA version 5, BIAevaluation Version 4.1, Flowjo (version 10)

For manuscripts utilizing custom algorithms or software that are central to the research but not yet described in published literature, software must be made available to editors and reviewers. We strongly encourage code deposition in a community repository (e.g. GitHub). See the Nature Research guidelines for submitting code & software for further information.

Data

Policy information about availability of data

All manuscripts must include a data availability statement. This statement should provide the following information, where applicable:

- Accession codes, unique identifiers, or web links for publicly available datasets
- A list of figures that have associated raw data
- A description of any restrictions on data availability

Sequence data has been deposited in the GenBank and available with accession no. MW218376-MW218394 and MW218395.

Field-specific reporting

Life sciences study design

All studies must disclose on these points even when the disclosure is negative.

Sample size	No statistical method was used to determine the sample size. The sample size of three independent biological replicates (n=3) for each condition in both tissue culture and animal experiments was chosen based on the field standards for sufficient reproducibility and statistical analysis.
Data exclusions	No data were excluded.
Replication	The experiments were replicated successfully at least three times for representative images and blots, with highly reproducible results.
Randomization	The animals were allocated randomly to experimental groups.
Blinding	Blinding was not relevant to the study because the results are quantitative, which are objective measures, and did not require a subjective judgment or based on qualitative scoring metrics. All experimental conditions are well controlled and appropriate controls were also done in parallel. No bias could be made by the tester in the experiments performed.

Reporting for specific materials, systems and methods

We require information from authors about some types of materials, experimental systems and methods used in many studies. Here, indicate whether each material, system or method listed is relevant to your study. If you are not sure if a list item applies to your research, read the appropriate section before selecting a response.

Materials & experimental systems

n/a	Involved in the study	
☐	☒	Antibodies
☐	☒	Eukaryotic cell lines
☒	☐	Palaeontology and archaeology
☐	☒	Animals and other organisms
☒	☐	Human research participants
☒	☐	Clinical data
☒	☐	Dual use research of concern

Methods

n/a	Involved in the study	
☒	☐	ChIP-seq
☐	☒	Flow cytometry
☒	☐	MRI-based neuroimaging

Antibodies

Antibodies used	6x-His Tag Monoclonal Antibody FITC, [AD1.1.10] (Invitrogen, MA1-81891), Alexa Fluor 647 conjugated anti-HA tag monoclonal antibody (Biolegend, Catalogue#682404), Dynabeads™ Goat Anti-Mouse IgG (Invitrogen, Catalogue#11033), Mouse anti-HA tag monoclonal antibody [HA.C5] (Abcam, Catalogue#ab18181), Goat anti-mouse HRP (Invitrogen, Catalogue#626520), Biotinylated anti-mouse IgG (Vector Laboratories, Catalogue#MKB-2225-.1), Goat Anti-Human IgG Fc (HRP) (Abcam, Catalogue#ab97225), FITC-conjugated goat anti-mouse monoclonal antibody (Invitrogen, Catalogue#62-6511), anti-HKU4 S1 monoclonal antibody (Cambridge Biologics, Catalogue#01-03-0117).
Validation	Validation statement for each primary antibody is provided on the manufacturer's website.

Eukaryotic cell lines

Policy information about cell lines

Cell line source(s)	Calu-3 (ATCC HTB-55), HeLa (ATCC CCL-2), HRT-18G (ATCC CRL-11663), HT-29 (ATCC HTB-38), A549 (ATCC CCL-185), Hep-2 (ATCC CCL-23), NT2/D1 (ATCC CRL-1973), RD (ATCC CCL-136), THP-1 (ATCC TIB-202), Raji (ATCC CCL-86), 293T (ATCC CRL-3216), Caco-2 (ATCC HTB-37), CRFK (ATCC CCL-94), PK15 (ATCC CCL-33), 3T3 (ATCC CCL-92), RK3E (ATCC CRL-1895), LLC-MK2 (ATCC CCL-7), Vero (ATCC CCL-81), Vero E6 (ATCC CRL-1586), BS-C-1 (ATCC CCL-26), Huh-7 (JCRB0403), NHBE (Lonza CC-2540), Caki-3-R (CCLV-RIE 1284), His-1 (in house development), HFL (in house development), Dubca (ATCC CRL-2276), Sf9 (ATCC CRL-1711), HPK (Pomona roundleaf bat), MPK (Lesser bent-winged bat), MRK (Rickett's big-footed bat), MRL (Rickett's big-footed bat), PAK (Japanese pipistrelle), PAL (Japanese pipistrelle), RSK (Chinese horseshoe bat), RSL (Chinese horseshoe bat), TPK (Lesser bamboo bat), TPL (Lesser bamboo bat), RLK(Leschenault's rousette), RLL (Leschenault's rousette). All bat cell lines are in house developed.
Authentication	Cells were not subjected to additional cell authentication.
Mycoplasma contamination	All cell lines were negative for mycoplasma contamination.
Commonly misidentified lines (See ICLAC register)	No commonly misidentified cell lines were used.

Animals and other organisms

Policy information about studies involving animals; ARRIVE guidelines recommended for reporting animal research

Laboratory animals	6-8 weeks old female hDPP4 transgenic mice (C57BL/6N) were involved in the study.
Wild animals	Bats were involved in the study, they were captured by both harp traps and mist nets. Samples were taken from the bats at the site where they were captured. Bats were released at the same spot right after the samples collection was completed.
Field-collected samples	The study did not involve samples collected from the field.
Ethics oversight	Department of Agriculture, Fisheries and Conservation, HKSAR; Committee on the Use of Live Animals in Teaching and Research, The University of Hong Kong and Department of Health, HKSAR.

Note that full information on the approval of the study protocol must also be provided in the manuscript.

Flow Cytometry

Plots

Confirm that:

- [x] The axis labels state the marker and fluorochrome used (e.g. CD4-FITC).
- [x] The axis scales are clearly visible. Include numbers along axes only for bottom left plot of group (a 'group' is an analysis of identical markers).
- [x] All plots are contour plots with outliers or pseudocolor plots.
- [x] A numerical value for number of cells or percentage (with statistics) is provided.

Methodology

Sample preparation	For detecting MERS-RBD-Fc-His and HKU4-RBD-Fc-His, cells were stained with 6x-His Tag Monoclonal Antibody FITC (MA1-81891, Invitrogen) at 1:10 dilution for 30 min. After washing cells with PBS-1% BSA, cells were then fixed by 4% PFA. For surface staining of DPP4s (hDPP4-HA, TpDPP4-HA, and dcDPP4-HA), cells were then stained with Alexa Fluor 647 conjugated anti-HA antibody (682404, Biolegend) at 1:500 dilution in 1% BSA-PBS for 30 min in the dark and washed with PBS-1% BSA.
Instrument	BD FACSCantoII Analyzer
Software	Flow cytometry data was collected with BD FACS Diva software and analyzed with FlowJo (version 10)
Cell population abundance	No sorting was performed with the flow cytometer
Gating strategy	Gating strategies were described in Supplementary Table 8 and Supplementary Fig. 5. Gating strategy to determine the boundary of different DPP4 receptor-positive (Receptor+) cells was carried out using untransfected 293T cells whereas gating strategy to determine the boundary of RBD positive (RBD+) cells was carried out using Receptor+ cells.

- [x] Tick this box to confirm that a figure exemplifying the gating strategy is provided in the Supplementary Information.
